# Thyroid-associated Ophthalmopathy

**DOI:** 10.4274/tjo.80688

**Published:** 2017-04-01

**Authors:** Esra Şahlı, Kaan Gündüz

**Affiliations:** 1 Ankara Numune Training and Research Hospital, Ophthalmology Clinic, Ankara, Turkey; 2 Ankara University Faculty of Medicine, Department of Ophthalmology, Ankara, Turkey

**Keywords:** Thyroid ophthalmopathy, proptosis, steroid therapy, Radiotherapy, decompression surgery

## Abstract

Thyroid-associated ophthalmopathy is the most frequent extrathyroidal involvement of Graves’ disease but it sometimes occurs in euthyroid or hypothyroid patients. Thyroid-associated ophthalmopathy is an autoimmune disorder, but its pathogenesis is not completely understood. Autoimmunity against putative antigens shared by the thyroid and the orbit plays a role in the pathogenesis of disease. There is an increased volume of extraocular muscles, orbital connective and adipose tissues. Clinical findings of thyroid-associated ophthalmopathy are soft tissue involvement, eyelid retraction, proptosis, compressive optic neuropathy, and restrictive myopathy. To assess the activity of the ophthalmopathy and response to treatment, clinical activity score, which includes manifestations reflecting inflammatory changes, can be used. Supportive approaches can control symptoms and signs in mild cases. In severe active disease, systemic steroid and/or orbital radiotherapy are the main treatments. In inactive disease with proptosis, orbital decompression can be preferred. Miscellaneous treatments such as immunosuppressive drugs, somatostatin analogs, plasmapheresis, intravenous immunoglobulins and anticytokine therapies have been used in patients who are resistant to conventional treatments. Rehabilitative surgeries are often needed after treatment.

## INTRODUCTION

Thyroid-associated ophthalmopathy (TAO) is an ocular condition that frequently manifests with thyroid dysfunction, and is the most common extrathyroidal manifestation of Graves’ disease. Graves’ disease is an autoimmune disease characterized by hyperthyroidism, diffuse goiter, ophthalmopathy, and in rare cases, dermopathy. Thyroid dermopathy consists of pretibial cutaneous nodules or diffuse thickening. In addition to elevated free thyroid hormone levels and suppressed thyroid stimulating hormone (TSH) levels, the levels of serum antithyroglobulin (TG) antibodies, antithyroid peroxidase (anti-TPO), and TSH receptor antibodies levels may be elevated in Graves’ disease. Graves’ disease is the most common cause of hyperthyroidism.^1^ The annual incidence is 0.3% in the United States of America, 2.7% in women in the United Kingdom, and 0.3% in men in the United Kingdom. It is 6 to 7 times more common in females than males. It occurs more often in the 3^rd^ and 4^th^ decades.^2^ Although TAO is usually seen in patients with Graves’ disease (80%), it may also occur in patients with thyroid cancers or autoimmune hypothyroid due to Hashimoto’s thyroiditis (10%), as well as individuals with no thyroid disease (10%).^3^

### Epidemiology and Pathogenesis

While TAO is 2.5- to 6-fold more common among women, severe ophthalmopathy is more common among men. Onset is generally between the ages of 30 and 50, and the disease course is more severe after age 50. Ophthalmopathy is reported to occur in 25-50% of patients with Graves’ disease and 2% of patients with Hashimoto’s thyroiditis. About 3-5% of these patients have severe ophthalmopathy.^4^ Most patients develop ophthalmopathy within 18 months of being diagnosed with Graves’ disease. However, ophthalmopathy onset may occur up to 10 years before and as late as 20 years after the onset of thyroid disease.^5^

Although the pathogenesis of TAO is not completely understood, it is known to be an autoimmune disorder. It has been established that autoimmunity develops against antigens common to the thyroid gland and the orbit. Although some support the view that the common pathogenetic antigen is TSH receptor,^6^ Salvi et al.^7^ identified a 64-kDa protein common to the thyroid gland and the orbit. Recent studies have reported upregulation of the cardiac calsequestrin gene in TAO patients and suggested that autoimmunity against calsequestrin may be a triggering factor in the pathogenesis of ophthalmopathy.^8^ Despite a close correlation between ophthalmopathy and TSH receptor antibodies, soon after the publication of autoimmunity against calsequestrin, autobodies against orbital fibroblast membrane antigen collagen XIII were also identified.^9^

Reactive T lymphocytes that recognize thyroid-orbit common antigens infiltrate the orbit and extraocular muscle perimysium. This is enhanced by circulating and local adhesion molecules stimulated by cytokines. Following infiltration of the orbit with T lymphocytes, the common antigen is recognized by T-cell receptors on CD4+ T lymphocytes (Th). Cytokines secreted by Th lymphocytes activate CD8+ lymphocytes and autoantibody-producing B cells, which strengthens the immune reaction.^10^ These cytokines stimulate the synthesis and secretion of glycosaminoglycans (GAGs) by fibroblasts. Due to their water attracting properties, GAGs lead to periorbital edema, proptosis, and swelling of the extraocular muscles.^11^ Fibroblast proliferation stimulated by cytokines also plays a role in the expansion of the orbital contents. Orbital fibroblasts include preadipocytes, which turn into adipocytes with hormonal stimulation. These cells have been shown to contribute to the increase in the volume of retroorbital fat tissue.^12^

Recent studies have demonstrated that thyroid autoantibodies and immune system genes have an important role in predicting before the development of ophthalmopathy and determining its severity after onset. Anti-TPO antibody and anti-TG positivity rates of 90% and 50%, respectively, have been reported in the presence of ophthalmopathy.^13,14^

In addition to autoimmunity, genetic and environmental factors are also known to be influential in the etiopathogenesis of thyroid ophthalmopathy.

### Genetic Factors

There are many studies investigating the role of genetics in the development of ophthalmopathy. In a study evaluating the ocular and palpebral findings of first and second degree relatives of patients with TAO, Graves’ disease, and Hashimoto’s thyroiditis, TAO findings such as upper eyelid retraction were present in 33% of euthyroid relatives.^15^ Twin studies have shown that the frequency of Graves’ disease is up to 30% in monozygotic twins, and it has been predicted that the risk of developing Graves’ disease is influenced approximately 79% by genetics and 21% by environmental factors.^16^

Many studies have reported polymorphisms in protein genes affecting immune function such as HLADR-3, CTLA 4, PTPN22, CD40, interleukin (IL)-2RA, FCRL3, and IL-23R, as well as genes encoding thyroid-specific proteins like TG.^17^

The presence of single-nucleotide polymorphisms (SNPs) in the genes of tyrosine phosphatase, which affects TSH receptor, and the genes of inflammatory cytokines IL-13, IL-21, and IL-23 has been demonstrated in TAO patients.^18,19,20,21,22^ Gene polymorphism for transcription regulator NF-κB1 has been associated with the development and onset age of ophthalmopathy.^23^ A study evaluating the relationship between major histocompatibility complex (MHC) class II human leukocyte antigen (HLA) alleles and ophthalmopathy revealed an association between the HLA-DRB1 allele and extraocular muscle involvement.^24^ SNPs identified in the ARID5B and NRXN3 genes may also regulate fat deposition and have a link to Graves’ disease.^25,26^ It has been shown that a nucleotide substitution in a TG gene promoter associated with interferon alpha (IFNα) was more common in patients with autoimmune thyroid disease. The authors stated that IFNα directly affected gene expression underlying thyroid autoimmunity via the binding of IFN regulatory factor-1 to the variant TG promoter.^27^ In a recent study, calsequestrin-1 gene SNP was proposed as a genetic marker for TAO.^28^

### Environmental Factors

In individuals with the relevant genes, ophthalmopathy may be triggered by environmental factors such as stress, infectious agents, iodine, IFN and interleukin therapy, and sex steroids. Bacteria may trigger an inflammatory response either by stimulating the expression of costimulatory molecules like MHC class II or by altering presentation of their own proteins. Although there are reports in the literature linking Graves’ disease to human foamy virus and *Yersinia enterocolitica* infection, causal relationships could not be demonstrated.^17^ Cigarette use is the strongest modifiable risk factor. In fact, the risk is proportionate to the number of cigarettes smoked daily.^29^ TAO is more common and more severe in smokers, and smokers also relapse more often and more severely after treatment. Cawood et al.^30^ demonstrated that GAG production and adipogenesis increased in a dose-dependent manner in response to cigarette smoke extract in an in vitro TAO model. Moreover, smoking leads to delayed and reduced response to ophthalmopathy treatment.^31^

### Clinical Course and Signs

Patient evaluation begins with confirming the clinical diagnosis and determining the current disease phase; finally, determining the clinical severity is necessary in order to choose appropriate treatment.

Ophthalmic findings are usually bilateral, but may also be unilateral or asymmetric. Nearly half of Graves’ disease patients have symptoms including dryness and stinging, photophobia, epiphora, diplopia, and a feeling of pressure behind the eyes.^29^ In a study evaluating 120 TAO patients, the most common ocular findings were eyelid retraction (91%), proptosis (62%), extraocular muscle dysfunction (42%), conjunctival hyperemia (34%), eyelid edema (32%), and chemosis (23%). Findings of optic neuropathy were rarer (6%). In the same patient series, the most common symptom was diplopia (33%), followed by pain and discomfort (30%), epiphora (21%), photophobia (16%), and blurred vision (9%).^31^

Subclinical involvement is present in approximately 70% of patients with Graves’ hyperthyroidism. Expansion of the extraocular muscles may be apparent on magnetic resonance imaging (MRI) and computed tomography (CT). In approximately 3-5% of patients, the disease follows a severe course with severe pain, inflammation, sight-threatening corneal ulceration, and compressive optic neuropathy.^29^

The clinical manifestations of TAO can be evaluated under the headings of soft tissue inflammation, eyelid retraction, proptosis, restrictive myopathy, and optic neuropathy.

### Soft Tissue Inflammation

Soft tissue inflammation is often the earliest sign of TAO. Soft tissue involvement consists of periorbital edema, conjunctival hyperemia, chemosis, and superior limbic keratoconjunctivitis (SLK). Symptoms may include foreign body sensation, epiphora, palpebral and conjunctival hyperemia and edema, blurred vision, and retroorbital pain. Periorbital edema may lead to prolapse of the retroseptal adipose tissue into the eyelid, venous circulatory disturbance, and retroseptal infiltration. SLK is characterized by upper tarsal conjunctival papillae, superior bulbar conjunctival hyperemia, limbal papillary hypertrophy, punctate epitheliopathy, and filaments in the upper cornea. Thyroid function tests should be performed for all patients with SLK.

### Eyelid Retraction

**Figure 1:**
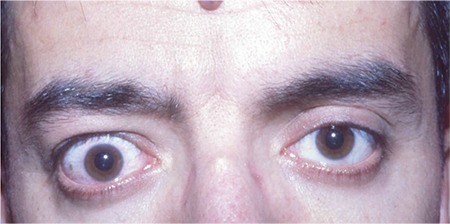
Right upper lid retraction in a 38-year-old male patient. Upper lid retraction (Dalrymple’s sign) may be one of the initial signs of thyroid-associated ophthalmopathy

Upper eyelid retraction (Dalrymple’s sign) may emerge as an early sign of TAO. Upper eyelid retraction in TAO may be caused by increased sympathetic stimulation of Müller’s muscle by thyroid hormone, but may also be attributed to the formation of scar tissue between the levator muscle and surrounding tissues, or to overaction of the levator muscle contracting against a tight inferior rectus muscle (Figure 1).^29^ In addition to upper eyelid retraction, upper eyelid lag (von Graefe’s sign) is also an important sign. Upper eyelid lag refers to a delay in the upper eyelid following as the eye rotates downward as a patient tracks an moving object. This is also an important criterion in the early diagnosis of TAO.

### Proptosis

**Figure 2:**
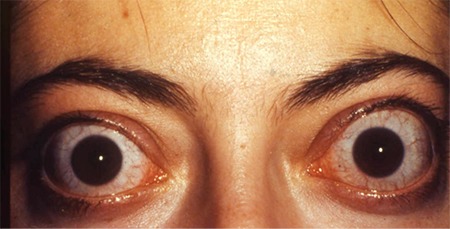
Bilateral infiltrative thyroid-associated ophthalmopathy in a 33-year-old female patient. Hertel exophthalmometer values were 28 mm for both eyes

Proptosis is spontaneous decompression resulting from enlargement of the extraocular muscles and adipose tissue, as well as orbital fat deposits and the infiltration of orbital tissues by GAGs and leukocytes (Figure 2). TAO is the most common cause of unilateral and bilateral proptosis in adults. It does not respond to hyperthyroidism treatment, and is permanent in 70% of cases. Proptosis is usually (90%) bilateral. Complications such as exposure keratopathy, corneal ulcer, and even corneal perforation may occur in cases of severe proptosis due to the eyelids not fully closing. Upper eyelid retraction may be confused with proptosis. Conditions producing pseudoproptosis include conditions in which the eyeball is enlarged, such as degenerative myopia and congenital glaucoma (buphthalmos), upper eyelid retraction, and contralateral enophthalmos.

### Restrictive Myopathy

**Figure 3:**
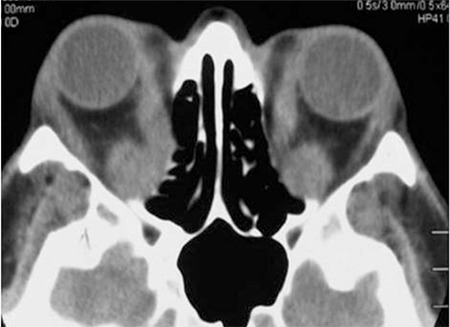
Orbital computed tomography images showing enlarged inferior and medial rectus muscles in a patient with thyroid-associated ophthalmopathy. The inferior rectus muscle is enlarged, mimicing an orbital tumor

**Figure 4:**
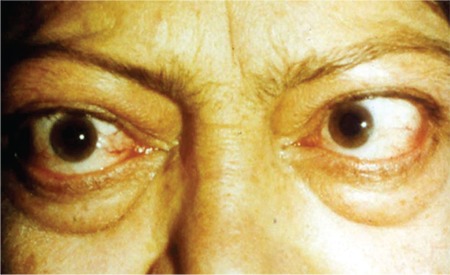
Internal rotation of the left eye due to fibrosis of the left medial rectus muscle in a 55-year-old patient with thyroid-associated ophthalmopathy

Eye movements are restricted due to edema that occurs in the extraocular muscles during the infiltrative stage and the subsequent fibrosis. Diplopia manifesting as the appearance of overlapping images is common. In primary and reading positions, it affects daily activities and causes patients significant discomfort. Despite expansion of the extraocular muscles in TAO, the muscle fibers themselves are normal. Muscle enlargement occurs due to separation of the muscle fibrils by fluid and fat deposits and by GAG material, fibrosis, scar formation, and leukocyte infiltration. Usually a single muscle is involved. While any of the six extraocular muscles may be involved, enlargement of the inferior rectus muscle is seen in most patients (Figure 3), followed by medial and superior rectus muscle involvement (Figure 4).^32^ Pressure exerted by a fibrotic inferior rectus muscle on the globe may cause a spike in intraocular pressure during upgaze. In some cases, extraocular muscle fibrosis may also be associated with chronically elevated intraocular pressure.^33^

### Optic Neuropathy

Optic neuropathy develops as a result of pressure from enlarged muscles on the optic nerve or the vessels that supply it. It may present with gradual decline in visual acuity, color vision disturbance, and central or paracentral scotomas. Fundus examination is usually normal, though optic disc edema, choroidal folds, optic disc paleness may be observed. The presence of optic neuropathy is often not correlated with proptosis.^34^

Orbital imaging may be done with ultrasound, CD, or MRI. Ultrasound allows rapid evaluating, but requires an experienced operator. CT and MRI have the advantage of imaging the entire orbit. CT is more sensitive for showing extraocular muscle enlargement. In active disease, the extraocular muscles appear as hyperintense on T2-weighted MRI.^35^

Although the natural course of ophthalmopathy is not fully understood, it has an inflammatory active phase that lasts an average of 3-6 months but may be as long as 3 years, followed by a fibrotic inactive phase. About 1% of patients experience reactivation after a period of inactivity. There is no indicator signaling the beginning of the inactive phase, but stability of clinical findings for a period of 6 months may indicate transition to inactive phase.^36^

In 1969, Werner^36^ first systematically classified the clinical characteristics of TAO in order to determine severity of ophthalmopathy. He divided the ocular findings by severity into seven classes, and named the classification system with the acronym “NOSPECS” based on the first letter of each class. The classification was modified in 1977 by the American Thyroid Association.^37^ It is not widely used today due to several limitations, including its reliance on subjective criteria, inability to assess disease activity, and the fact that the irregular clinical progression exhibited by most patients does not conform well to the classification system.

In 1989, Mourits et al.^38^ developed the Clinical Activity Score (CAS) for evaluating ophthalmopathy activity (Table 1). According to this formula, which includes 10 different inflammatory changes, each finding is scored to yield an activity score between 0 and 10. In 1992, a committee formed by four thyroid societies modified the CAS and reduced the number of criteria. The modified version was published to facilitate the evaluation of ocular changes following ophthalmopathy treatment (Table 2).^39^

According to the European Group on Graves’ Orbitopathy (EUGOGO), a CAS score of 3 or higher defines active TAO with a 65% positive predictive value for response to radiotherapy. According to this, it can be expected that patients with higher CAS values will respond better to treatment.^29,40^ Regardless, the CAS has certain limitations such as being dependent on the evaluator and being inadequate for following clinical changes.^41^

More recently, Dolman and Rootman^42^ developed the VISA classification, based on 4 findings: vision, inflammation, strabismus, and appearance (Table 3). Each parameter is separately graded and scored. Active disease is defined as worsening in any of the VISA parameters. Another classification system most commonly used in evaluating the activity and severity of TAO and making treatment decisions is the EUGOGO classification (Table 4).^43^

### Treatment

Most TAO patients have mild and nonprogressive ocular involvement which does not require treatment. Less severe ophthalmopathies tend to resolve spontaneously.^3^

Treatment options for TAO can be grouped into medical and surgical therapies. Medical treatment is appropriate for patients with active disease. These treatments are not effective for inactive ophthalmopathy and carry the risk of side effects. Surgical interventions can be implemented in cases where the threat to vision cannot be controlled with medical treatment, and in cases with inactive disease in order to protect function and improve appearance.

Because cigarette smoking increases the severity of ophthalmopathy and reduces treatment response, patients should be urged to quit smoking.^44^ Thyroid dysfunction, particularly hypothyroidism, negatively affects ophthalmopathy onset; therefore, a euthyroid state must be achieved as quickly as possible and maintained.^45^ Euthyroidism may be achieved with antithyroid drugs, radioactive iodine (RAI) therapy, or thyroidectomy. However, it has been shown that RAI therapy leads to new ophthalmopathy development and exacerbates existing ophthalmopathy. This effect does not occur with combined RAI and steroid therapy.^46^ The effect of RAI on ophthalmopathy may be explained by two mechanisms. Antigens common to the thyroid and retroorbital tissues may be released due to radiation-induced thyroid damage, and these antigens may play a role in the development of immune-mediated ophthalmopathy. Alternatively, RAI therapy may stimulate the secretion of TSH due to the rapid induction of hypothyroidism, thereby stimulating antigen production by thyrocytes.^47^ In contrast, a recent study reported that RAI therapy did not increase the risk of ophthalmopathy development or exacerbation.^48^

Topical lubricants are recommended to protect the cornea and alleviate symptoms of dryness. In addition to using artificial tear drops or gel during the day, at night the eyelids may be taped closed to prevent conjunctival exposure and ointments can be applied. Guanethidine and beta blocker eye drops can be used to treat eyelid retraction. Patients with pronounced periorbital edema may benefit from elevating the head at night. Wearing sunglasses may also provide symptomatic relief. Prismatic spectacles may be prescribed to patients with diplopia.^49^ Botulinum toxin injection may provide temporary improvement in upper lid retraction and restrictive myopathy.^50,51^

## MEDICAL TREATMENT

### Steroid Therapy

Steroids are still the best medical treatment for active TAO. In addition to their anti-inflammatory and immunosuppressive effects, they also reduce the synthesis and secretion of GAG by orbital fibroblasts. Steroids may be administered via oral, intravenous, retrobulbar, and subconjunctival routes. Retrobulbar and subconjunctival application of steroids is not commonly performed due to side effects and lack of efficacy.^49^

For oral steroids to be effective, high doses (60-100 mg/day or higher prednisolone) and long duration (10-20 weeks) are usually required. Based on treatment response in the first few weeks, the initial dose can be gradually reduced. A decrement of 5-10 mg per week has been shown to be generally safe. However, some patients experience recurrence when medication is reduced or discontinued. Medical treatment has been shown to be effective for soft tissue changes, ocular motility, and optic neuropathy, but its effect on proptosis is limited. Following high-dose steroid therapy, some patients who require steroid treatment again due to trauma, surgery, or infection may develop adrenal insufficiency. Treatment is limited to a few months in patients exhibiting side effects such as Cushingoid appearance, diabetes, hypertension, and osteoporosis. If long-term therapy is required, using nonsteroid immunosuppressants or orbital radiotherapy as supplemental treatment allows the steroid dose to be reduced.^52^

**Figure 5:**
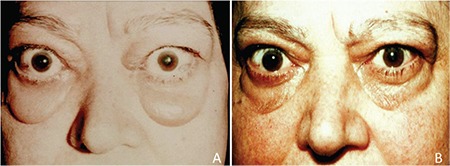
A 61-year-old female patient with infiltrative thyroid-associated ophthalmopathy. The patient exhibted significant palpebral and conjunctival edema and reported severe pain (A). The same patient showed substantial regression of clinical signs after 3 months of intravenous corticosteroid therapy (B)

Intravenous steroids are often administered at a high dose (0.5-1 g methyl prednisolone) for 3 days, followed by oral prednisolone. Giving intravenous pulse steroids in 1- or 2-week cycles has been determined more effective than oral steroids (Figure 5A, 5B). Studies comparing the efficacy of oral and intravenous steroids have reported that intravenous steroids are superior in reducing CAS, and that steroid-related side effects such as Cushingoid appearance, diabetes, hypertension, osteoporosis, and gastric irritation are more common with oral steroids.^52^ In a controlled study comparing intravenous and oral steroid therapy, Kahaly et al.^53^ compared a group that received intravenous 500 mg methyl prednisolone once a week for 6 weeks, followed by 250 mg methyl prednisolone once a week for 6 weeks (total 4.5 g) with a group that received oral prednisolone starting at 100 mg/day and reduced by 10 mg per week over 12 weeks (total 4 g). Treatment response was defined as reductions in proptosis, palpebral aperture, ocular pressure, and rectus muscle width; improvement in diplopia; and increase in visual acuity. The authors reported that 77% of patients in the intravenous group and 60% of patients in the oral group responded to treatment. During 6 months of follow-up, the group that received oral steroids required surgical intervention and exhibited optic neuropathy more often. Macchia et al.^54^ conducted a study comparing treatment with intravenous 1 g methyl prednisolone twice a week for 6 weeks and treatment with oral prednisolone starting at 60-80 mg/day and reduced every other week over 4-6 months. In both groups, there was marked reduction in orbital inflammation symptoms and findings, and substantial improvement in proptosis and diplopia. The oral steroid group exhibited side effects related to treatment. In another study, 3-day pulse 1 g methyl prednisolone followed by 3-month oral prednisolone therapy was compared with oral prednisolone therapy alone, and no differences in diplopia, proptosis, or soft tissue activity score were found.^55^ An advantage of pulse intravenous steroid therapy is that treatment response may be seen within 1-2 weeks. Intravenous steroids are most effective in reducing inflammatory soft tissue findings and ocular motility dysfunction. However, rare cases that developed acute, severe liver failure during pulse steroid therapy have been documented. The cumulative methyl prednisolone dose in these cases was 10-24 g. This acute liver damage was found to be associated with previous viral hepatitis. Sudden discontinuation of intravenous steroid therapy may exacerbate underlying autoimmune liver disease. Therefore, it is advisable to limit the cumulative methyl prednisolone dose to 6-8 mg and identify patients at risk by evaluating liver morphology, viral markers, and autoantibodies prior to treatment.^52,56,57^

### Orbital Radiotherapy

Orbital radiotherapy is used in the management of ophthalmopathy due to its nonspecific anti-inflammatory effects, its reduction of GAG production, and the high radiosensitivity of the lymphocytes that infiltrate orbital tissue.^58^ The main benefit of orbital radiation therapy is the improvement of ocular motility. In the treatment of ophthalmopathy, orbital radiotherapy (cumulative dose of 20 Gy in 10 divided fractions) was shown to be equivalent to placebo in terms of activity score, proptosis, and lid retraction, and superior to placebo in the correction of diplopia.^59^ In general, treatment is administered as 1500-2000 cGy divided over 10 days. A study comparing the efficacy of high- and low-dose radiation (16 Gy versus 2.4 Gy and 20 Gy versus 10 Gy) reported no marked difference in effect and stated that the radiotherapy dose should not exceed 2.4 Gy for TAO.^60^ It may take a few weeks for the effect of radiation to become apparent; the effect is temporary and may cause an increase in inflammation. Therefore, steroid therapy should be continued in the first few weeks of treatment. The effect is gradual initially and reaches a peak after 6 months. The main side effect is early onset cataract. It may also cause radiation retinopathy and radiation-induced optic neuropathy. These complications are not common when the dose is properly divided and the eyes are closed. Marquez et al.^61^ followed patients for an average of 11 years after radiation therapy and determined a 12% rate of cataract development. Diabetes is considered a relative contraindication for radiation due to the risk of exacerbating retinopathy.^62^

It was reported that combined orbital radiotherapy and systemic steroid therapy was substantially more effective than radiotherapy or steroid therapy alone.^63^ Although orbital radiotherapy and oral steroid therapy have similar efficacy, side effects are reported to occur more often with steroids.^64^

### Immunosuppressive Therapies

Due to the autoimmune mechanism of TAO, various immunosuppressive drugs such as cyclosporin, azathioprine, and cyclophosphamide, as well as immunomodulatory agents like ciamexon have been used in treatment. Cyclosporin is the most commonly used immunosuppressive drug in the treatment of ophthalmopathy. Cyclosporin acts by inhibiting cytotoxic T lymphocyte activation and antigen presentation by monocyte and macrophages, which in turn activates suppressor T lymphocytes and inhibits cytokine production. Compared to oral steroid therapy alone, supplementation of oral steroid therapy with cyclosporin results in a greater reduction in activity score, provides marked improvement in proptosis and diplopia, and reduces the relapse rate after discontinuation of steroid therapy.^65^ There is no consensus on the efficacy of azathioprine therapy in ophthalmopathy. Despite a reduction in thyroid-associated antibodies in patients receiving azathioprine, no difference was observed in clinical parameters compared to a control group.^66^ There are case reports in the literature demonstrating clinical and immunologic improvement with intravenous cyclophosphamide in patients resistant to steroid therapy and/or radiotherapy.^67,68,69^

Treating ophthalmopathy with agents such as octreotide, pentoxifylline, nicotinamide, plasmapheresis, and intravenous immunoglobulin (IVIg) has been attempted, but these are not among the main treatment methods.

### Somatostatin Analogues

Octreotide is a synthetic somatostatin analogue, and octreoscan-111 positivity may reflect TAO activity and be predictive of treatment response.^70^ In a study conducted in France, a reduction in CAS was observed in patients treated with extended-release octreotide. Despite a significant reduction in proptosis, the authors reported that octreotide was not effective in mitigating the activity of mild TAO.^71^ Although there are some cases in which octreotide resulted in improvement in soft tissue findings, many studies have demonstrated that it is not adequately effective.^70,72,73^ Due to octreotide’s short halflife, the long-acting somatostatin analogue lanreotide was developed. Lanreotide administered every other week for 3 months was shown to be effective in the treatment of ophthalmopathy, particularly soft tissue findings.^74^

### Pentoxifylline and Nicotinamide

The beneficial effects of pentoxifylline and nicotinamide in the treatment of ophthalmopathy have been demonstrated in a limited number of studies. Both agents are believed to act by inhibiting fibroblast GAG synthesis induced cytokines. Compared to a control group, pentoxifylline was reported to be effective in reducing inflammatory symptoms and correcting proptosis.^75,76^

### Intravenous Immunoglobulin

The role of plasmapheresis and IVIg in the treatment of ophthalmopathy has yet to be definitively determined. While some studies have shown that IVIg therapy has a similar effect to oral steroid therapy and radiotherapy in patients with active TAO,^77^ Seppel et al.^78^ reported that IVIg therapy was ineffective against ophthalmopathy.

### Plasmapheresis

Plasmapheresis aims to remove the immunoglobulins and immunocomplexes involved in TAO pathogenesis. When performed in combination with immunosuppressive therapy in 4 sessions within a period of 5-8 days, significant improvement was noted in clinical signs of ophthalmopathy; however, after 1 year, recurrence occured in some patients and treatment was repeated.^79^ Plasmapheresis may be used as a last resort for severe TAO when all other treatments have failed.

### Anticytokine and Antilymphocyte Antibodies

Anticytokine and antilymphocyte monoclonal antibodies are a new therapeutic approach which may be applied in patients who do not respond to conventional immunosuppressive therapies.^68^ There are studies in the literature demonstrating the efficacy of anti-tumor necrosis factor alpha (anti-TNFα) monoclonal antibodies (etanercept, infliximab), anti-CD-25 antibody (daclizumab), and anti-B lymphocyte antibody (rituximab) against inflammatory symptoms of ophthalmopathy.^69,80,81^ Targeting TNF may affect the production of chemoattractant protein 1 and macrophage-attracting protein by preadipocytes in TAO.29 The monoclonal antibody rituximab, which inhibits active B cells, seems promising.^82^ A study demonstrated that rituximab therapy resulted in a significant decrease in the stimulatory anti-thyrotropin receptor antibody subgroup.^83^

Topical 5% guanethidine drops were previously used to treat upper eyelid retraction, but is not used in contemporary practice.^84^

### Surgical Therapy

Approximately 5% of TAO patients require surgical intervention. Necessary procedures should be performed in the following order: orbital decompression, strabismus surgery, lid lengthening surgery, and blepharoplasty.

**Figure 6:**
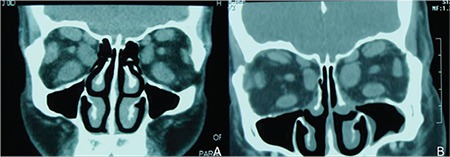
Coronal computed tomography of a patient with thyroid-associated ophthalmopathy (A). Coronal computed tomography images from the same patient after orbital decompression surgery (B). Postoperative images show the absence of the medial orbital wall and thinning of the cortical bone in the lateral wall

Orbital decompression surgery consists of enlarging the bony orbit, extracting orbital adipose tissue, or a combination of the two. Indications for the procedure are compressive optic neuropathy that does not respond to steroid therapy or orbital radiotherapy, or prominent proptosis which will lead to severe corneal involvement.^1^ It should not be performed during the active stage of the disease. A recent randomized, controlled study compared surgical versus medical decompression as initial treatment in cases with optic neuropathy and showed that urgent decompression surgery did not yield better outcomes than steroid therapy. In the presence of optic neuropathy, the first choice of treatment should be intravenous followed by oral steroid therapy.^85^ The goal of decompression surgery is to increase the volume of the bony orbit, thereby directly relieving apical pressure as much as possible. Commonly used techniques are removal of the medial and inferior wall, removal of the inferomedial and lateral wall, balanced removal of the medial and lateral wall, and deep lateral wall decompression. Although decompression can be achieved through the medial orbital wall, force applied by the retractors can increase the already high retrobulbar pressure and exceed a critical level for the optic nerve fibers. Preventative removal of the lateral wall facilitates access to the deep orbit and reduces the risk of elevated orbital pressure. Transcaruncular or inferior fornix approaches in medial wall removal prevent scar formation. The endoscopic transnasal approach is an alternative that provides apical access without increasing intraorbital floor.^86^ The main disadvantage of the antral-ethmoidal decompression with transantral approach described in 1957 by De Santo^87^ is the resulting motility dysfunction in 52% of cases. For patients with moderate exophthalmos, antral-ethmoidal decompression via the eyelid is a valid alternative due to the low risk of iatrogenic diplopia (4.6%). With more severe exophthalmos, combined inferomedial decompression and lateral decompression may be performed.^86^ In 1989, Leone et al.^88^ recommended balanced removal of the medial and lateral walls in order to reduce strabismus after decompression. Although this technique was considered to theoretically reduce the risk of iatrogenic diplopia, the risk was determined to be higher than that in removal of the lateral wall alone or with the inferomedial wall, as well as 3-wall removal.^89^ Medial wall, orbital floor, and lateral wall removal continue to be preferred in contemporary bony decompression surgery (Figure 6A, 6B). Removal of the orbital roof is no longer practiced because it contributes minimally to orbital enlargement and carries the risk of potential complications and side effects. Minimally invasive approaches and hidden incisions in the conjunctiva or at the upper eyelid fold are preferred. According to exophthalmos severity, lateral wall decompression and/or adipose tissue removal, especially from the inferolateral quadrant, may be performed in addition to inferomedial decompression. To decrease postoperative diplopia, lateral wall removal with or without fat excision is recommended first, followed by medial and inferior wall removal if necessary.^86^ Recently, deep lateral wall removal has been described as a part of a rehabilitative 3-wall decompression with coronal approach. A 32% reduction in exophthalmos was reported with this technique, without increasing the risk of consecutive diplopia compared to conventional 3-wall decompression. However, some claim that the volume of the deep lateral wall is highly variable between individuals and may not always provide sufficient orbital volume.^90^

A different approach to decompression of the orbital contents involves removing orbital fat in addition to medial or inferolateral orbitotomy. A mean reduction in proptosis of 1.8 mm (0-6 mm) was reported using this technique.^91^ Combining fat removal with bony decompression has gained popularity in recent years, and is superior to either fat or bone removal alone in terms of safety and effectiveness.^86^

Fat removal orbital decompression confers greater risk of damaging the oculomotor nerve ciliary branch, lacrimal nerve, orbital vasculature, extraocular muscles, optic nerve and globe than bony decompression. Rare complications of bony decompression include consecutive strabismus; infraorbital hypoesthesia; sinusitis; lower lid entropion; cerebrospinal fluid leakage; central nervous system infections; damage to the globe, optic nerve, or vasculature; cerebral vasospasm; ischemia; and infarct.^86^ Another rare (1.3%) complication reported in recent years is TAO reactivation following rehabilitative bony decompression. This phenomenon is characterized by active TAO symptoms and findings emerging a few weeks after a normal postoperative recovery period in patients not under perioperative steroid therapy and was named ‘delayed decompression-related reactivation’. It is treated with systemic immunosuppression or radiotherapy.^92^

Extraocular muscle surgery is performed to correct diplopia. The disease should be stable for 6 months. The muscle that most often requires corrective surgery is the inferior rectus, followed by the medial rectus. Adjustable sutures should be preferred. This reduces the need for multiple operations, which is not uncommon. Surgeries involving more than one muscle should be avoided and recession procedures should be preferred over resection in order to prevent ocular ischemic syndrome. Factors which may lead to surgical failure are tightness and hemorrhagic tendency of the extraocular muscles, potential postoperative scarring, and restricted access to the surgical area due to lid edema.^93^ Strabismus surgery is necessary for most patients with severe ophthalmopathy to restore binocular single vision in primary position and while reading.

Eyelid surgery is performed as an emergency procedure (tarsorrhaphy) in patients with exposure keratitis or corneal ulcer, or often for rehabilitation and in cases of lid malformation. Patients should be euthyroid and ophthalmopathy should be stabile and inactive for 6-12 months before surgery. Müller’s muscle excision or recession is often adequate for treating upper lid retraction. Recession of the levator aponeurosis or levator myotomy may be performed.^94^ Lower lid retraction may be corrected by recession of the lid retractors with the insertion of acellular dermal, tarsal, or conjunctival spacer material.^95^

The treatment plan for TAO should be determined individually for each patient. Timely diagnosis is critical for patients at risk for developing serious complications like restrictive myopathy and optic neuropathy. High-risk patients (such as older patients, males, diabetics, and smokers), those with family history of ophthalmopathy, and patients with moderate inflammatory signs should be followed closely. Urgent interventions are necessary for patients with color or central vision loss, progressive diplopia, or severe inflammatory signs.^43^

In light of the current literature, management of TAO may follow the following algorithm: Euthyroidism should be achieved in all patients with TAO and they should be strongly encouraged to quit if they smoke cigarettes. In cases of mild disease, treatments and practices such as topical lubricants, wearing sunglasses, elevating the head during sleep, and using prismatic glasses or botulinum toxin injection to Müller’s muscle for diplopia may provide symptomatic relief. With moderate and severe disease, most patients show improvement in inflammatory soft tissue changes and muscle motility dysfunction with intravenous steroid therapy. For patients who do not respond to steroid therapy, immunosuppressive therapies (oral steroids combined with cyclosporin or cyclophosphamide) are an option, and orbital radiotherapy may be preferred for patients with pronounced ocular motility dysfunction. Antilymphocyte antibody (rituximab) may be tried in patients who do not respond to conventional immunosuppressive therapy. In moderate and severe inactive disease, orbital decompression surgery, strabismus surgery, recession of the levator or lid retractors, and blepharoplasty may be performed as necessary in the specified order. For active disease with sight-threatening severe exposure keratopathy, severe proptosis, or compressive optic neuropathy, patients who do not respond to intravenous pulse steroid therapy followed by oral steroid therapy or orbital radiotherapy are candidates for urgent orbital decompression surgery. Patients with severe corneal involvement may also benefit from procedures such as lateral tarsorrhaphy, amniotic membrane transplantation, and keratoplasty.^43^

## CONCLUSION

TAO is an autoimmune disease with significant impact on quality of life. Although in most patients ophthalmopathy is mild and nonprogressive, it is of the utmost importance that patients at risk be followed closely and treated appropriately and in a timely manner based on disease severity and activity.

## Figures and Tables

**Table 1:**
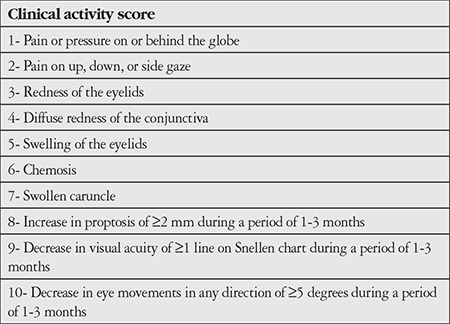
Clinical activity score criteria. Active disease is accepted as the presence of 3 or more of the first 7 criteria for patients not examined within the previous 3 months, or 4 or more of the 10 criteria for patients examined within the previous 3 months (Mourits MP, Koornneef L, Wiersinga WM, Prummel MF, Berghout A, van der Gaag R. Clinical criteria for the assessment of disease activity in Graves’ ophthalmopathy: a novel approach. Br J Ophthalmol. 1989;73:639-644.)

**Table 2:**
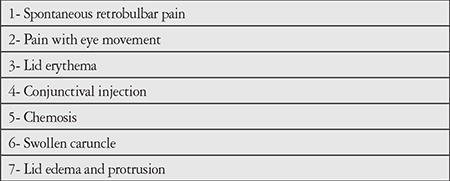
Modified clinical activity score criteria (Pinchera A, Wiersinga W, Glinoer D, Kendall-Taylor P, Koornneef L, Marcocci C, Schleusener H, Romaldini J, Niepominiscze H, Nagataki S, Izumi M, Inoue Y, Stockigt J, Wall J, Greenspan F, Solomon D, Garrity J, Gorman CA. Classification of eye changes of Graves’ disease. Thyroid. 1992;2:235-236.)

**Table 3:**
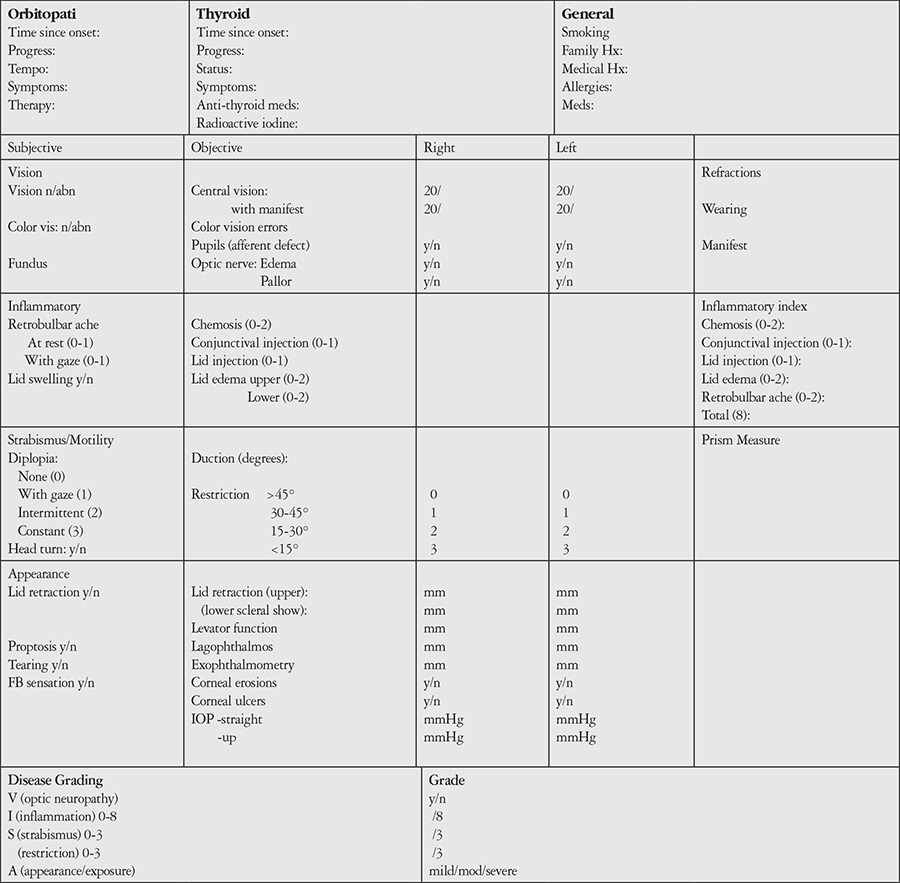
VISA classification (Dolman PJ, Rootman J. VISA classification for Graves orbitopathy. Ophthal Plast Reconstr Surg. 2006;22:319-324)

**Table 4:**
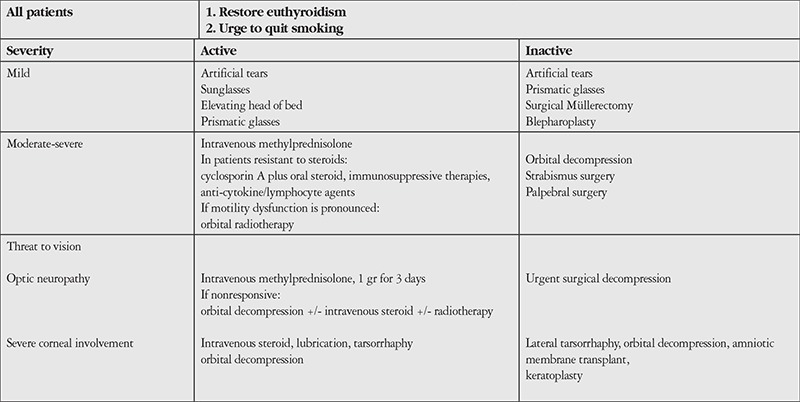
Treatment algorithm for thyroid-associated ophthalmopathy (Barrio-Barrio J, Sabater AL, Bonet-Farriol E, Velázquez-Villoria Á, Galofré JC. Graves’ ophthalmopathy: VISA versus EUGOGO classification, assessment and management. J Ophthalmol. 2015;2015:249125)
